# The New Pharmacopœia

**Published:** 1882-12-20

**Authors:** 


### *THE NEW PHARMACOPŒIA.*

The U. S. Pharmacopœia, of 1880, has been issued. Dr. Squibb, in the November issue of his Ephemeris, refers to the increased strength of the opium preparations as matter important to be borne in mind by the druggist and the practitioner.

As an example, if the full dose of the deodorized tincture of opium (com. solution of opium) has heretofore been 24 minims, or 38 drops, representing a quarter of a grain of sulphate of morphine, the corresponding dose of the new preparations will be 16 minims or 25 drops.

This makes a pound of the new preparation equal to a pound and a half of the old. Dr. Squibb suggests that in physicians' prescriptions, where, for example, deodorized tincture of opium is prescribed, the figures 1870 or 1880 be added to indicate the strength desired until such time as the change becomes established.

